# Impact of NDUFAF6 on breast cancer prognosis: linking mitochondrial regulation to immune response and PD-L1 expression

**DOI:** 10.1186/s12935-024-03244-1

**Published:** 2024-03-08

**Authors:** Baohong Jiang, Sixuan Wu, Lijun Zeng, Yuanbin Tang, Lunqi Luo, Lianjie Ouyang, Wenjie Feng, Yeru Tan, Yuehua Li

**Affiliations:** 1 Department of Pharmacy, The First Affiliated Hospital, Hengyang Medical School, University of South China, Hengyang, Hunan People’s Republic of China; 2 Department of Oncology, The First Affiliated Hospital, Hengyang Medical School, University of South China, Hengyang, Hunan People’s Republic of China; 3 Clinical Oncology School of Fujian Medical University, Fujian Cancer Hospital, Fuzhou, Fujian China

**Keywords:** Breast cancer, NDUFAF6, NRF2, PD-L1, Immune infiltration, Prognosis

## Abstract

**Background:**

Breast cancer is a major global health concern, and there is a continuous search for novel biomarkers to predict its prognosis. The mitochondrial protein NDUFAF6, previously studied in liver cancer, is now being investigated for its role in breast cancer. This study aims to explore the expression and functional significance of NDUFAF6 in breast cancer using various databases and experimental models.

**Methods:**

We analyzed breast cancer samples from The Cancer Genome Atlas (TCGA), Gene Expression Omnibus (GEO), and Human Protein Atlas (HPA) databases, supplemented with immunohistochemistry (IHC) staining to assess NDUFAF6 expression. A breast cancer cell xenograft mouse model was used to evaluate tumor growth, apoptosis, and NDUFAF6 expression. Survival probabilities were estimated through Kaplan–Meier plots and Cox regression analysis. A Protein–Protein Interaction (PPI) network was constructed, and differentially expressed genes related to NDUFAF6 were analyzed using GO, KEGG, and GSEA. The relationship between NDUFAF6 expression, immune checkpoints, and immune infiltration was also evaluated.

**Results:**

NDUFAF6 was found to be overexpressed in breast cancer patients and in the xenograft mouse model. Its expression correlated with worse clinical features and prognosis. NDUFAF6 expression was an independent predictor of breast cancer outcomes in both univariate and multivariate analyses. Functionally, NDUFAF6 is implicated in several immune-related pathways. Crucially, NDUFAF6 expression correlated with various immune infiltrating cells and checkpoints, particularly promoting PD-L1 expression by inhibiting the NRF2 signaling pathway.

**Conclusion:**

The study establishes NDUFAF6 as a potential prognostic biomarker in breast cancer. Its mechanism of action, involving the inhibition of NRF2 to upregulate PD-L1, highlights its significance in the disease's progression and potential as a target for immunotherapy.

**Supplementary Information:**

The online version contains supplementary material available at 10.1186/s12935-024-03244-1.

## Introduction

Breast cancer (BC) remains one of the leading causes of cancer-related deaths among women [1, 2]. According to a recent report, BC's estimated incidence and mortality cases in 2021 were 284,200 and 44,130, respectively [3]. BC is a multifaceted disease characterized by differential expression of numerous molecular biomarkers and genetic, epigenetic, and phenotypic alterations [4, 5]. BC is associated with risk variables such as family history, aging, and high breast density [6, 7]. Certain subtypes of BC are likely to develop resistance to chemotherapy, leading to very poor therapeutic outcomes presenting a major hurdle for clinicians aiming to maximize patient survival [8]. Metastatic BC is typically inoperable, with treatments limited to chemotherapy, hormone therapy, targeted therapy, etc., with suboptimal efficacy [9, 10]. Among the strategies to combat the BC epidemic, emphasis has been placed on prevention, early diagnosis, and drug treatment [11]. Thus, identifying new reliable diagnostic biomarkers and establishing unique BC immunotherapeutic targets is paramount.

The NDUFAF6 gene is an assembly factor for the NADH ubiquinone oxidoreductase complex [6]. Previously considered c8orf38, it was first shown to be associated with mitochondrial function in 2008 [11, 12]. The encoded protein is localized to the mitochondrial inner membrane and, besides playing a crucial role in the assembly of Complex I (CI) of the mitochondrial respiratory chain, it controls the synthesis of the mtDNA-encoded protein ND1 [13]. The human NDUFAF6 gene is associated with Leigh syndrome, an early-onset severe neuro-metabolic disorder [14, 15]. This gene mutation leads to CI enzyme deficiency or Arcadia variant of Fanconi syndrome [16, 17]. Recently, NDUFAF6 was found to have significant prognostic potential in hepatocellular carcinoma [18]. However, the expression and clinical significance of NDUFAF6 in BC remain unclear.

Immune infiltration is pivotal in the tumor microenvironment and is closely related to tumor growth, metastasis, and treatment response [19]. PD-L1 is a crucial immune checkpoint, and its expression on tumor cells is associated with immune evasion and poor prognosis [20]. In recent years, inhibitors of PD-L1 and its receptor PD-1 have emerged as effective treatments for various solid tumors, including breast cancer [21, 22]. However, the molecular mechanisms regulating PD-L1 expression remain largely elusive.

Against this backdrop, we delved into the role of NDUFAF6 in breast cancer using the Cancer Genome Atlas (TCGA) and Gene Expression Omnibus (GEO) databases. We propose that NDUFAF6 is an independent negative prognostic biomarker for BC patients, as listed in online databases such as TCGA. Additionally, we analyzed the differential genomic alterations and functional networks associated with NDUFAF6 expression and its role in tumor immunity. Finally, we validated the key mechanisms using an in vivo animal model. This discovery provides additional evidence supporting the hypothesis of NDUFAF6 as a therapeutic target or predictive biomarker and offers new theoretical foundations and practical guidance for the diagnosis, treatment, and prognosis assessment of breast cancer.

## Materials and methods

### Clinical data collection

In exploring the expression and function of the mitochondrial protein NADH-ubiquinone oxidoreductase complex assembly factor 6 (NDUFAF6) in BC, we initially relied on the Cancer Genome Atlas (TCGA) database. We extracted RNA-Seq data concerning NDUFAF6 in BC, specifically utilizing 1083 sample instances processed with HTSeq-FPKM and HTSeq-Counts. To ensure data quality and reliability, we excluded samples lacking RNA sequencing data or with an overall survival (OS) of less than 30 days. Based on the obtained HTSeq-FPKM data, we further computed normalized expression values of transcripts, namely transcripts per million reads. To delve deeper into the function and expression pattern of NDUFAF6 in BC, we divided the samples into high and low NDUFAF6 expression groups. Using the DESeq2 R package, we analyzed differential gene expression, setting significance thresholds at |log2 fold change (FC)|> 1.5 and adjusted p-value ≤ 0.05.

To validate our findings based on TCGA, we further utilized datasets GSE109169 and GSE22820 from the Gene Expression Omnibus (GEO), providing an independent validation set for our results. Beyond public database resources, we also conducted on-site laboratory research. We obtained tissue samples from BC patients from the First Affiliated Hospital of South University and Hengyang Medical College. Using the Human Protein Atlas (HPA) database and immunohistochemistry (IHC) techniques, we further assessed the local expression pattern of NDUFAF6 in tissue samples. Throughout these studies, we strictly adhered to ethical guidelines, ensuring all lab research received approval from the relevant ethics committee and consistently followed the requirements of the Declaration of Helsinki. By integrating database resources and laboratory techniques, we conducted a thorough and systematic exploration of the function and expression of NDUFAF6 in BC.

### Tissue microarray construction and immunohistochemical expression assessment

To delve deeper into the expression of NDUFAF6 in BC, we employed tissue microarray technology, preparing samples from malignant and benign tissues of BC patients. Each tissue sample underwent paraffin fixation. Based on this, we collected 1.5 mm diameter tissue cores fixed in formalin and subsequently processed with paraffin, from which we prepared 4 µm thick tissue sections. These 4-µm sections were then used for tissue microarray construction.

For immunohistochemical (IHC) analysis, we used a specific NDUFAF6 antibody (bs-19077R) diluted to a 1:100 concentration. As a secondary antibody, we chose one conjugated with horseradish peroxidase, sourced from Dako Cytomation (Carpinteria, CA, USA). We enlisted two experienced pathologists to evaluate the staining results. They employed the following scoring criteria: negative (0 points), weakly positive (1 point), moderately positive (2 points), and strongly positive (3 points). Additionally, based on the proportion of positive cells in the sample, further scoring was conducted: 0–10% (1 point), 11–50% (2 points), 51–80% (3 points), and 81–100% (4 points). Combining the two criteria, a total score of 4 or above was defined as a high expression, while scores below 4 were considered low.

### Kaplan–Meier plot modeling and performance assessment

We constructed a Kaplan–Meier plot based on the multivariate Cox regression analysis results. By summing the weights of each parameter in the predictive model, a composite risk score was assigned to each patient. To evaluate the accuracy of this model, we used calibration plots and observed a high concordance between the bias-corrected line and the 45-degree line of perfect calibration, indicating predictions matched observed risks. Further, we assessed the discriminative ability of the Kaplan–Meier plot by calculating the concordance index (C-index) and employing a bootstrap resampling method with 1,000 iterations. All statistical tests were two-sided, with a significance level set at p ≤ 0.05.

### PPI network-based NDUFAF6 co-expressed gene protein interactions and functional enrichment analysis

First, we used online database search tools to predict NDUFAF6 co-expressed genes and constructed their protein–protein interaction (PPI) network to obtain functional interaction information between proteins. Using Cytoscape software (version 3.8), we further filtered the top 10 genes with the strongest interaction with NDUFAF6, where only interactions with a combined score above 0.4 were considered statistically significant. Next, to identify differentially expressed genes (DEGs), we used the DESeq2 package (version 3.8) in R to compare expression patterns between low and high NDUFAF6 expression groups, employing the Wilcoxon rank-sum test for differential analysis. In filtering DEGs, we set thresholds at |log2 fold change (FC)|> 1.5 and adjusted p-value ≤ 0.05. Finally, to explore these DEGs' biological functions and metabolic pathways, we conducted GO and KEGG pathway enrichment analyses using the ClusterProfiler software package.

### GSEA

Gene Set Enrichment Analysis (GSEA) is an advanced computational method aimed at determining whether a specific gene set shows significant expression differences between two distinct biological states (e.g., disease vs. normal) [23]. To explore the functional role of NDUFAF6 in breast cancer and its associated biological pathways, we conducted GSEA analysis comparing high and low NDUFAF6 expression groups, utilizing the ClusterProfiler package in R. To ensure result reliability, we performed 1,000 permutation tests. When filtering for significant functions or pathways, we set thresholds at an adjusted p-value ≤ 0.05 and a false discovery rate (FDR) < 0.25.

### Correlation analysis of immune cell infiltration

The single-sample gene set enrichment analysis (ssGSEA) is a commonly used method for immune cell infiltration analysis. This method compares the gene expression data of each sample with specific immune cell gene sets to estimate the relative enrichment levels of immune cell gene sets in the sample. In immune cell infiltration analysis, we utilized ssGSEA to assess the degree of infiltration of 24 different immune cells in cancer tissues. The analysis settings were optimized for ssGSEA, focusing on normalization and scaling to accurately assess the degree of immune cell infiltration, followed by Spearman correlation analysis to investigate the relationship between the expression of NDUFAF6 and the degree of immune cell infiltration. To visually describe the strength of these associations, we defined the absolute value of the correlation coefficient according to the following criteria: 0.00 to 0.05 as extremely weak correlation; 0.06 to 0.10 as weak correlation; 0.11 to 0.15 as moderate correlation; greater than 0.15 as strong correlation. In all analyses, a p-value ≤ 0.05 was considered statistically significant. Finally, we compared the differences in the expression of the NDUFAF6 gene among the 24 tumor-infiltrating immune cells by plotting a line graph.

### Association analysis of NDUFAF6 expression with stromal, immune and ESTIMATE scores

To systematically evaluate the matrix components, immune activity, and overall tissue microenvironment in BC samples, we employed the "ESTIMATE" method to compute stromal scores, immune scores, and ESTIMATE scores. These scores provided us with quantitative information about the various components of the tumor microenvironment. Further, to explore the correlation between NDUFAF6 expression and the scores above, we used the "limma" package in R in conjunction with the "ESTIMATE" tool. This analysis aimed to reveal the potential role and associations of NDUFAF6 in the BC microenvironment.

### R tool analysis of NDUFAF6 co-expression with immune checkpoint genes

To delve deeper into the potential associations between NDUFAF6 and immune checkpoint genes, we utilized the "limma" package in R for differential expression analysis. Further, we employed the "reshape2" package for data restructuring to fit subsequent analysis workflows. To visually display the co-expression patterns between NDUFAF6 and immune checkpoint genes, we used the "RColorBrewer" package for high-quality color mapping and visualization. This series of analyses aimed to explicitly reveal the role of NDUFAF6 in immune regulation and its relationships with major immune checkpoint genes.

### Cell culture and group transfection

The MCF-7 human breast cancer cell line was obtained from the American Type Culture Collection (ATCC). The cells were cultured in MEM medium (Gibco, Carlsbad, CA) supplemented with 0.01 mg/ml recombinant human insulin, 1% penicillin–streptomycin (Invitrogen), and 10% fetal bovine serum (FBS, Gibco). The cells were maintained in a cell culture incubator at 37 °C with 5% CO_2_. Upon reaching 80% confluence, the cells were dissociated and passaged using 0.25% trypsin/EDTA.

Cells were divided into the following groups: (1) ov-NC group (transfected with empty vector); (2) ov-NDUFAF6 group (transfected with NDUFAF6 overexpression vector); (3) sh-NC group (transfected with shRNA control vector); (4) sh-NDUFAF6 group (transfected with shRNA vector targeting NDUFAF6); (5) ov-NRF2 group (transfected with NRF2 overexpression vector). For transfection, logarithmically growing MCF-7 cells (1 × 10^6^) were seeded into 6-well plates, 2 mL medium per well, and transfected when cell confluence reached 50%.

Lipofectamine 3000 transfection reagent (Thermo Fisher Scientific) was used following the manufacturer's protocol for transfections. NDUFAF6 expression vector, NRF2 overexpression vector, or empty vector (all purchased from Shanghai Hanheng Biotechnology Co., Ltd., Shanghai, China) were separately transfected into MCF-7 cells. ShRNA vectors targeting NDUFAF6 and control shRNA vectors were used for transfection for the shRNA groups. As cells grew, once cell confluence reached 80%, we began selection using puromycin (Invitrogen, A1113803) to obtain successfully transfected stable cell lines. Forty-eight hours post-transfection, the efficiency of NDUFAF6 expression or knockdown was verified through Western blot.

### Establishment of nude mouse xenograft model

Female Balb/c nude mice (6–8 weeks old) were purchased from Beijing Vital River Laboratory Animal Technology Co., Ltd. (401). They were housed in separate cages in an SPF-grade animal laboratory with a humidity of 60 ~ 65% and a temperature of 22 ~ 25 °C. After a week of acclimatization feeding, experiments were initiated. The health status of the mice was observed before the experiment. The relevant ethics committee approved the experimental procedures and animal use protocols.

To establish the in vivo model, MCF-7 cells (1.0 × 10^6^) were subcutaneously injected into the mammary fat pad of the nude mice, forming experimental and control groups. All mice were divided into the following 4 groups for treatment and observation: (1) ov-NC group (injected with MCF-7 cells transfected with an empty vector); (2) ov-NDUFAF6 group (injected with MCF-7 cells transfected with the NDUFAF6 overexpression vector); (3) sh-NC group (injected with MCF-7 cells transfected with shRNA control vector); (4) sh-NDUFAF6 group (injected with MCF-7 cells transfected with shRNA vector targeting NDUFAF6). Subsequently, to validate the role of NRF2 in the model, all mice were divided into 2 groups for treatment and observation: the ov-NC group and the ov-NRF2 group. Finally, the growth of the tumors in the mice was observed, and when the tumor volume reached approximately 500 mm^3^, the mice were euthanized, and the tumor tissues were used for subsequent experimental tests. Concurrently, tumor volume changes were periodically recorded using measuring tools during the experiment.

### Western blotting

First, we lysed the samples digested and collected from tumor tissues of each group with enhanced RIPA Lysis Buffer containing protease inhibitors (Wuhan Boster Biological Technology). The protein concentration was measured using a BCA protein assay kit (Wuhan Boster Biological Technology) to ensure equal protein loading. Subsequently, protein samples were separated via SDS-PAGE and then electro-transferred onto PVDF membranes. On the PVDF membranes, we blocked with 5% BSA at room temperature for 1 h to prevent non-specific binding, then added diluted primary antibodies, including β-actin (ab8226, 1/5000, Abcam), NDUFAF6 (ab110244, 1/1000, Abcam), NRF2 (SAB4501984, 1/1000, Sigma), and PD-L1 (ab213480, 1/1000, Abcam) and incubated overnight at 4 °C for specific protein binding. Afterward, the PVDF membrane was washed three times with PBST for 5 min each, then incubated with Anti-Mouse-HRP secondary antibody (Cat # 7076, 1/5000, CST) or Anti-Rabbit-HRP secondary antibody (Cat # 7074, 1/5000, CST) at room temperature for 1 h. Finally, an appropriate amount of ECL working solution (EMD Millipore, USA) was added to the transferred membrane and incubated at room temperature for 1 min. Excess ECL reagent was removed, sealed with plastic wrap, placed in a dark box, and exposed to X-ray film for 5–10 min, followed by development and fixation.

### Immunohistochemistry

Tissue specimens were fixed with 4% paraformaldehyde for 12 h, followed by paraffin embedding and sectioning at a thickness of 3 μm. Sections underwent routine xylene deparaffinization and gradient alcohol hydration (absolute ethanol, 95% ethanol, 75% ethanol, each for 3 min). Antigen retrieval was performed by boiling in 0.01 M citrate buffer for 15–20 min. Sections were then incubated in 3% H2O2 at room temperature for 30 min to quench endogenous peroxidase activity. A goat serum-blocking solution was applied, and after 20 min, excess liquid was removed. Sections were then incubated with 50 μl of ki-67 primary antibody (ab15580, 1/100; Abcam) at room temperature for 1 h. After washing with PBS, sections were incubated with goat anti-rabbit IgG secondary antibody (ab6721, 1/100; Abcam) at 37 °C for 20 min, followed by SP (streptavidin-peroxidase) at 37 °C for 30 min. DAB (ST033, Weijia Biotechnology Co., Ltd., Guangzhou, China) was applied for 5–10 min for color development, followed by counterstaining with hematoxylin (PT001, Bogoo Biotechnology Co., Ltd., Shanghai, China) for 2 min. After differentiation with hydrochloric acid alcohol and washing for 10 min, sections underwent gradient alcohol dehydration and xylene clearing. The neutral resin was used for mounting. Observations and counts were made under an upright microscope (BX63, Olympus, Japan), and the average optical density of images was analyzed using Image-Pro Plus 6.0 software.

### TUNEL staining

Mouse tumor tissues were fixed with 4% paraformaldehyde for 15 min, washed three times with PBS, and permeabilized with 0.1% Triton-X 100 in PBS for 3 min. TUNEL staining was performed on the ovarian cancer tissue cells using a TUNEL staining kit (C1091, Beyotime, China). 50 μl of the biotin-labeled solution was added to the samples and incubated at 37 °C in the dark for 60 min. After washing with PBS three times, 50 μl of Streptavidin-HRP working solution was added and incubated at room temperature for 30 min. After three washes with PBS, 0.5 ml of DAB coloring solution was added and incubated at room temperature for 5 min. After three washes with PBS, the samples were counterstained with DAPI (10 μg/ml, C1025, Beyotime, Nantong, China) for 10 min. Images from each group were observed under a confocal microscope (FV1000, OLYMPUS), and the apoptosis ratio of each group of cells was calculated using Image Pro Plus 6.0 software.

### Statistical analysis

In this study, we first employed descriptive statistics to summarize the central tendency and distribution of the data. For continuous variables, depending on the data distribution, we used either the t-test or the Mann–Whitney U test for comparisons. The chi-square test or Fisher's exact test analyzed relationships between categorical variables. To assess the survival of breast cancer patients, we employed Kaplan–Meier survival analysis and used the Log-rank test to compare survival curves between groups. The Cox proportional hazards regression model was also used to evaluate various factors associated with survival time. All statistical analyses were two-sided, with p ≤ 0.05 as the statistical significance threshold. All data processing and analyses were performed using R software (version 4.2.0).

## Results

### Significant overexpression of NDUFAF6 in breast cancer tissues and its diagnostic potential

With the increasing understanding of the molecular mechanisms of breast cancer, the role of gene expression differences in cancer onset and progression has gained attention. Studies have reported that targeting mitochondrial oxidative phosphorylation (OXPHOS) is an emerging therapeutic strategy for cancer, and high OXPHOS tumors demonstrate high expression of mitochondrial respiratory complex I (NADH-ubiquinone oxidoreductase complex I) at the protein and mRNA level. Inhibiting the activity of mitochondrial respiratory complex I can suppress tumor growth and proliferation [23–25]. Therefore, in this study, we first explored the expression patterns of members of the NADH-ubiquinone oxidoreductase complex I in breast cancer (BC). Using the TCGA database, we clearly demonstrated differential expression of members of the NADH-ubiquinone oxidoreductase complex I in both breast cancer tissues and unmatched healthy tissue samples, as well as the expression contrasts between breast cancer and their corresponding non-cancerous tissue samples (Additional file 1: Fig. S1, Additional file 2: Fig. S2). We found that a majority of complex I members showed differential expression in breast cancer patients, with NDUFAF3, NDUFAF8, NDUFS2, NDUFS3, NDUFS5, NDUFS6, NDUFS7 and NDUFAS8, and NDUFS8 being upregulated in breast cancer tissue, while NDUFAF4, MT-ND1 MT-ND2, MT-ND3, MT-ND4, MT-ND5, MT-ND6, and NDUFS4 were downregulated.

**Fig. 1:**
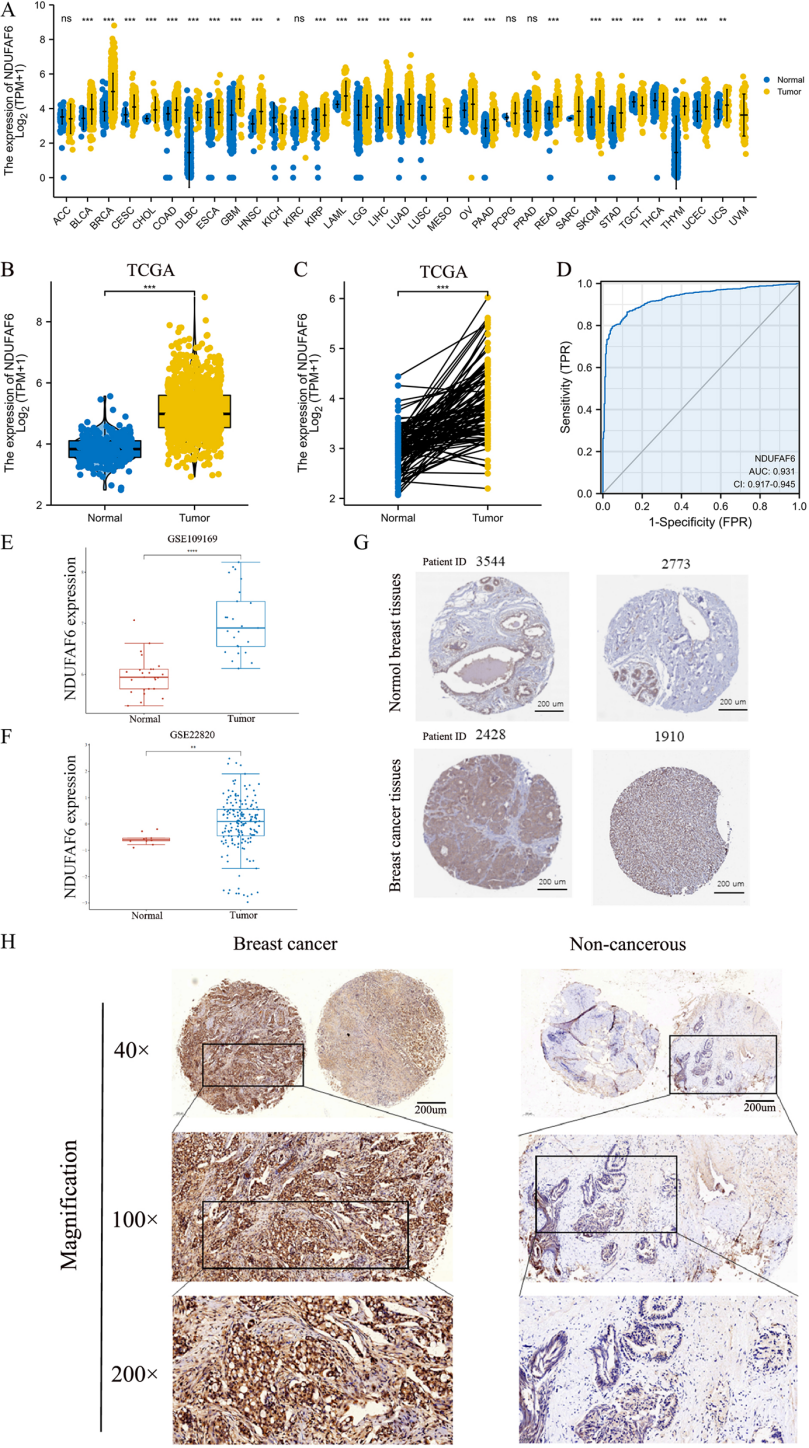
Differential expression of NDUFAF6 in breast cancer and healthy tissues and its potential value in tumor differentiation. **A** Distribution of NDUFAF6 expression across various cancer types and their matched healthy tissues. **B** Differential expression of NDUFAF6 in breast cancer tissues versus unmatched healthy tissue samples. **C** Comparison of NDUFAF6 expression in breast cancer and its corresponding non-cancerous tissue samples. **D** ROC curve analysis showcasing the diagnostic potential of NDUFAF6 in distinguishing breast cancer from healthy tissues. **E** Validation of NDUFAF6 expression pattern in the GEO database dataset GSE109169. **F** Further validation of NDUFAF6 expression in the GEO database dataset GSE22820. **G** Representative IHC micrographs of NDUFAF6 in breast samples and BC from the HPA database. **H** Representative IHC micrographs of NDUFAF6 in collected clinical samples of breast samples and BC

Specifically, the expression difference of NDUFAF6 between tumor and healthy tissue is shown in Fig. 1A. Furthermore, based on data from 1083 patients in TCGA (Table 1), we found that in 113 healthy tissue samples, the expression of NDUFAF6 was significantly lower than in cancer tissue (p < 0.001). Similarly, in 112 pairs of matched normal breast tissue and tumor tissue samples, the expression of NDUFAF6 was significantly increased in tumor tissue (Fig. 1B, C). It is worth noting that the expression of NDUFAF6 had a high discriminatory ability to distinguish cancer from healthy tissue, with an AUC value of 0.931 (Fig. 1D). To further validate our findings, we also referred to two datasets from the GEO database (GSE109169 and GSE22820), both of which supported our main conclusions (Fig. 1E, F). In order to confirm the higher expression of NDUFAF6 in BC at the tissue protein level compared to normal breast tissue, we used immunohistochemical results from the HPA database and collected clinical samples for immunohistochemistry experiments. The results showed that the protein expression level in BC tissue was significantly higher than in normal breast tissue. Typical microphotographs of IHC are shown in Fig. 1G, H. In conclusion, our study clearly demonstrates the significant overexpression of NDUFAF6 in breast cancer tissue, providing strong evidence for its potential as a therapeutic target for breast cancer.

**Table 1:** Clinical characteristics of the BC patients based on TCGA

Characteristic	Low expression of NDUFAF6	High expression of NDUFAF6	P value
n	541	542	
*T stage, n (%)*			0.944
T1	135 (12.5%)	142 (13.1%)	
T2	314 (29.1%)	315 (29.2%)	
T3	72 (6.7%)	67 (6.2%)	
T4	18 (1.7%)	17 (1.6%)	
*N stage, n (%)*			0.004
N0	271 (25.5%)	243 (22.8%)	
N1	186 (17.5%)	172 (16.2%)	
N2	40 (3.8%)	76 (7.1%)	
N3	39 (3.7%)	37 (3.5%)	
*M stage, n (%)*			0.130
M0	448 (48.6%)	454 (49.2%)	
M1	6 (0.7%)	14 (1.5%)	
*Pathologic stage, n (%)*			0.058
Stage I	90 (8.5%)	91 (8.6%)	
Stage II	325 (30.7%)	294 (27.7%)	
Stage III	109 (10.3%)	133 (12.5%)	
Stage IV	5 (0.5%)	13 (1.2%)	
*Race, n (%)*			0.043
Asian	22 (2.2%)	38 (3.8%)	
Black or African American	100 (10.1%)	81 (8.1%)	
White	387 (38.9%)	366 (36.8%)	
*Age, n (%)*			0.104
< = 60	314 (29%)	287 (26.5%)	
> 60	227 (21%)	255 (23.5%)	
*Histological type, n (%)*			0.001
Infiltrating ductal carcinoma	359 (36.7%)	413 (42.3%)	
Infiltrating lobular carcinoma	122 (12.5%)	83 (8.5%)	
*PR status, n (%)*			< 0.001
Negative	205 (19.8%)	137 (13.2%)	
Indeterminate	3 (0.3%)	1 (0.1%)	
Positive	309 (29.9%)	379 (36.7%)	
*ER status, n (%)*			< 0.001
Negative	162 (15.7%)	78 (7.5%)	
Indeterminate	0 (0%)	2 (0.2%)	
Positive	356 (34.4%)	437 (42.2%)	
*HER2 status, n (%)*			0.116
Negative	283 (38.9%)	275 (37.8%)	
Indeterminate	3 (0.4%)	9 (1.2%)	
Positive	71 (9.8%)	86 (11.8%)	
*PAM50, n (%)*			< 0.001
Normal	34 (3.1%)	6 (0.6%)	
LumA	294 (27.1%)	268 (24.7%)	
LumB	40 (3.7%)	164 (15.1%)	
Her2	46 (4.2%)	36 (3.3%)	
Basal	127 (11.7%)	68 (6.3%)	
*Menopause status, n (%)*			0.145
Pre	122 (12.6%)	107 (11%)	
Peri	23 (2.4%)	17 (1.7%)	
Post	331 (34.1%)	372 (38.3%)	
*Anatomic neoplasm subdivisions, n (%)*			0.288
Left	272 (25.1%)	291 (26.9%)	
Right	269 (24.8%)	251 (23.2%)	
*radiation_therapy, n (%)*			1.000
No	219 (22.2%)	215 (21.8%)	
Yes	280 (28.4%)	273 (27.7%)	
Age, median (IQR)	56 (48, 66)	59 (49, 68)	0.048

### Impact of NDUFAF6 expression on the growth of breast cancer cell xenografts

To delve deeper into the role of NDUFAF6 in breast cancer progression, we conducted a series of in vivo experiments to reveal its potential regulatory mechanisms on breast cancer cell proliferation and apoptosis.

**Fig. 2:**
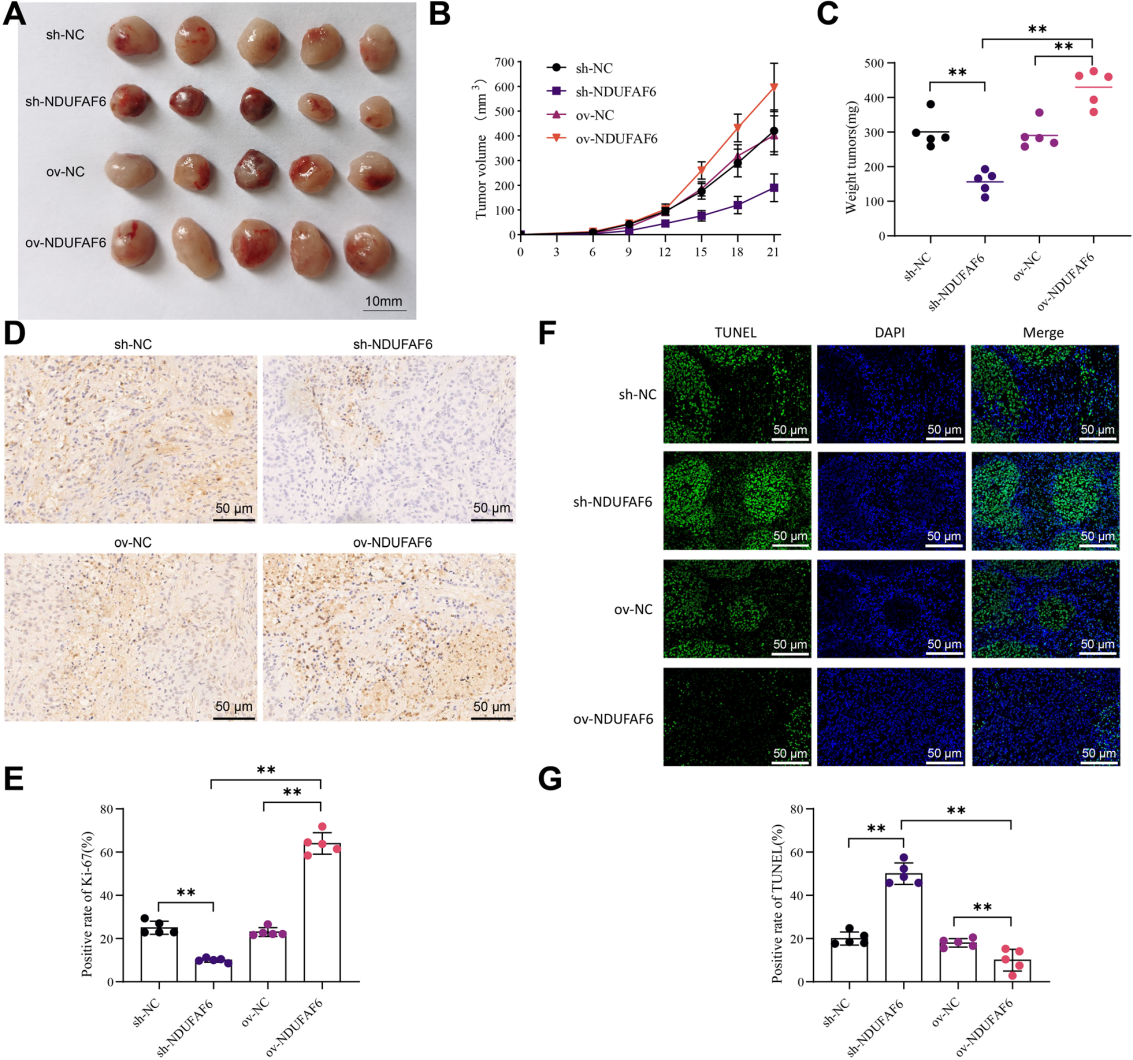
Impact of NDUFAF6 on the growth of MCF-7 cell xenograft in breast cancer. **A** Physical images of xenografts formed by MCF-7 cells (n = 5). **B** Impact of silencing or overexpressing NDUFAF6 on the volume of MCF-7 cell xenografts (n = 5). **C** Impact of silencing or overexpressing NDUFAF6 on the weight of MCF-7 cell xenografts and quantitative analysis (n = 5). **D**, **E** Immunohistochemical detection of Ki-67 changes and quantitative analysis of the Ki-67 positivity rate (n = 5). **F**, **G** Immunofluorescence detection of TUNEL changes and analysis of positivity rate (n = 5). **Indicates P < 0.01

We successfully established a breast cancer cell xenograft mouse model by subcutaneously inoculating MCF-7 breast cancer cells with inhibited or overexpressed NDUFAF6. Throughout the experiment, we continuously monitored and recorded the growth trends of these cell xenografts and the expression levels of NDUFAF6. Western Blot data revealed that compared to the sh-NC group, the expression of NDUFAF6 protein in the sh-NDUFAF6 group was significantly reduced (P < 0.01), whereas compared to the ov-NC group, the expression of NDUFAF6 protein in the ov-NDUFAF6 group was significantly increased (P < 0.01) (Additional file 3: Fig. S3). Further observations indicated that the downregulation of NDUFAF6 could significantly inhibit the growth of MCF-7 cell xenografts, mainly reflected in the volume and weight of the tumors (P < 0.01), while the upregulation of NDUFAF6 significantly accelerated the growth of the xenografts (Fig. 2A–C). To further decipher the biological functions of NDUFAF6, we measured the levels of Ki-67 protein in the xenografts as an indicator of cell proliferation. The results showed that accompanying the reduced expression of NDUFAF6, the proportion of Ki-67 positive cells significantly decreased, while the overexpression of NDUFAF6 led to an increase in the proportion of Ki-67 positive cells (P < 0.01) (Fig. 2D, E). Additionally, through TUNEL staining, we found that the downregulation of NDUFAF6 enhanced apoptosis in MCF-7 cell xenografts, while its upregulation inhibited cell apoptosis (P < 0.01) (Fig. 2F, G). Overall, NDUFAF6 significantly promoted the proliferation of breast cancer MCF-7 cells in vivo.

### Correlation analysis of NDUFAF6 expression with clinical pathological features in breast cancer

**Fig. 3:**
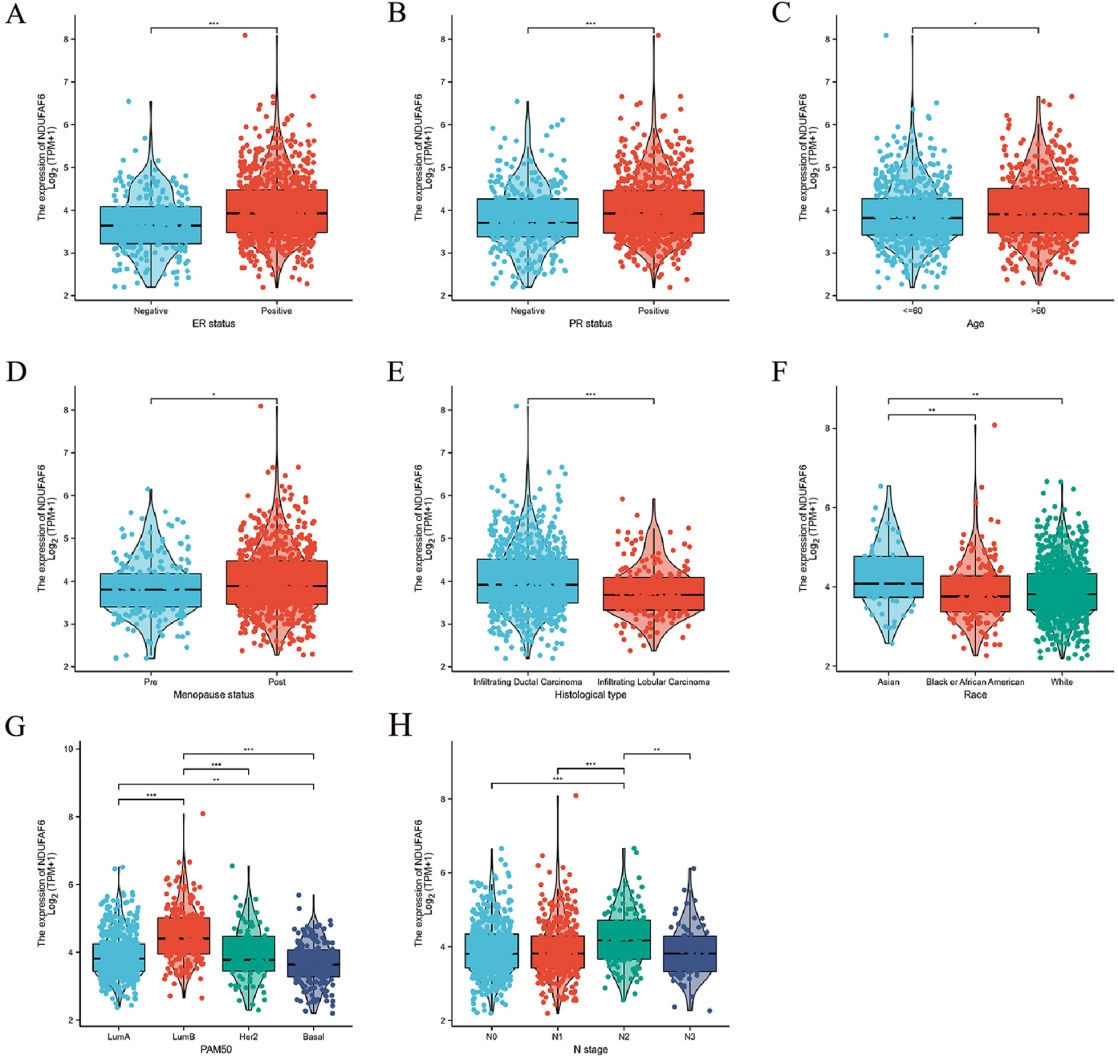
Association of NDUFAF6 expression in breast cancer with key clinical pathological parameters. **A** Correlation of NDUFAF6 expression with estrogen receptor (ER) status. **B** Correlation of NDUFAF6 expression with progesterone receptor (PR) status. **C** Association of NDUFAF6 expression with patient age. **D** Association of NDUFAF6 expression with menopausal status. **E** Relationship of NDUFAF6 expression with histological type. **F** Correlation of NDUFAF6 expression with patient race. **G** Association of NDUFAF6 expression with PAM50 subtyping. **H** Relationship of NDUFAF6 expression with N staging

In tumor biology research, identifying molecular biomarkers significantly associated with clinical pathological parameters holds decisive value for precision medicine and formulating individualized treatment strategies for patients. Against this backdrop, we focused on studying the expression pattern of the NDUFAF6 gene in breast cancer, especially its relationship with key clinical pathological indicators. Using the Wilcoxon rank-sum test, we distinctly revealed the close association of NDUFAF6 expression with ER status, PR status, age, menopausal status, and histological type (Fig. 3A–E). Further, Kruskal–Wallis rank-sum tests also confirmed its significant association with patient ethnicity, PAM50 subtype, and N staging (Fig. 3F–H).

Using logistic regression analysis, we further explored the relationship between NDUFAF6 expression and several adverse clinical features of breast cancer. Significant associations included its relationship with M stage, patient ethnicity, histological morphology, PR and ER status, and PAM50 subtype (Table 2).

**Table 2:** Logistic analysis of the relation between *NDUFAF6* expression and clinical variables in BC patients

Characteristics	Total (N)	Odds ratio (OR)	P value
T stage (T2&T3&T4 vs. T1)	1080	0.939 (0.714–1.234)	0.651
N stage (N1&N2&N3 vs. N0)	1064	1.199 (0.943–1.526)	0.139
M stage (M1 vs. M0)	922	2.302 (0.915–6.551)	0.090
Pathologic stage (Stage II&Stage III&Stage IV vs. Stage I)	1060	0.991 (0.719–1.366)	0.957
Race (Black or African American vs. Asian)	241	0.469 (0.254–0.849)	0.014
Age (> 60 vs. < = 60)	1083	1.229 (0.967–1.563)	0.092
Histological type (Infiltrating Lobular Carcinoma vs. Infiltrating Ductal Carcinoma)	977	0.591 (0.432–0.807)	< 0.001
PR status (Positive vs. Negative)	1030	1.835 (1.412–2.391)	< 0.001
ER status (Positive vs. Negative)	1033	2.549 (1.887–3.469)	< 0.001
HER2 status (Positive vs. Negative)	715	1.247 (0.874–1.782)	0.224
PAM50 (LumA&LumB&Her2 vs. Basal)	1043	2.300 (1.669–3.194)	< 0.001
Menopause status (Post vs. Pre)	932	1.281 (0.951–1.729)	0.104
Anatomic neoplasm subdivisions (Right vs. Left)	1083	0.872 (0.687–1.107)	0.261
Radiation_therapy (Yes vs. No)	987	0.993 (0.772–1.277)	0.957

These comprehensive analyses firmly establish the significant association between NDUFAF6 expression in breast cancer and several crucial clinical pathological parameters, strongly suggesting NDUFAF6 as a potential molecular biomarker in breast cancer patient's diagnosis, prognosis, and treatment.

### Prognostic value and survival analysis of NDUFAF6 expression in breast cancer

**Fig. 4:**
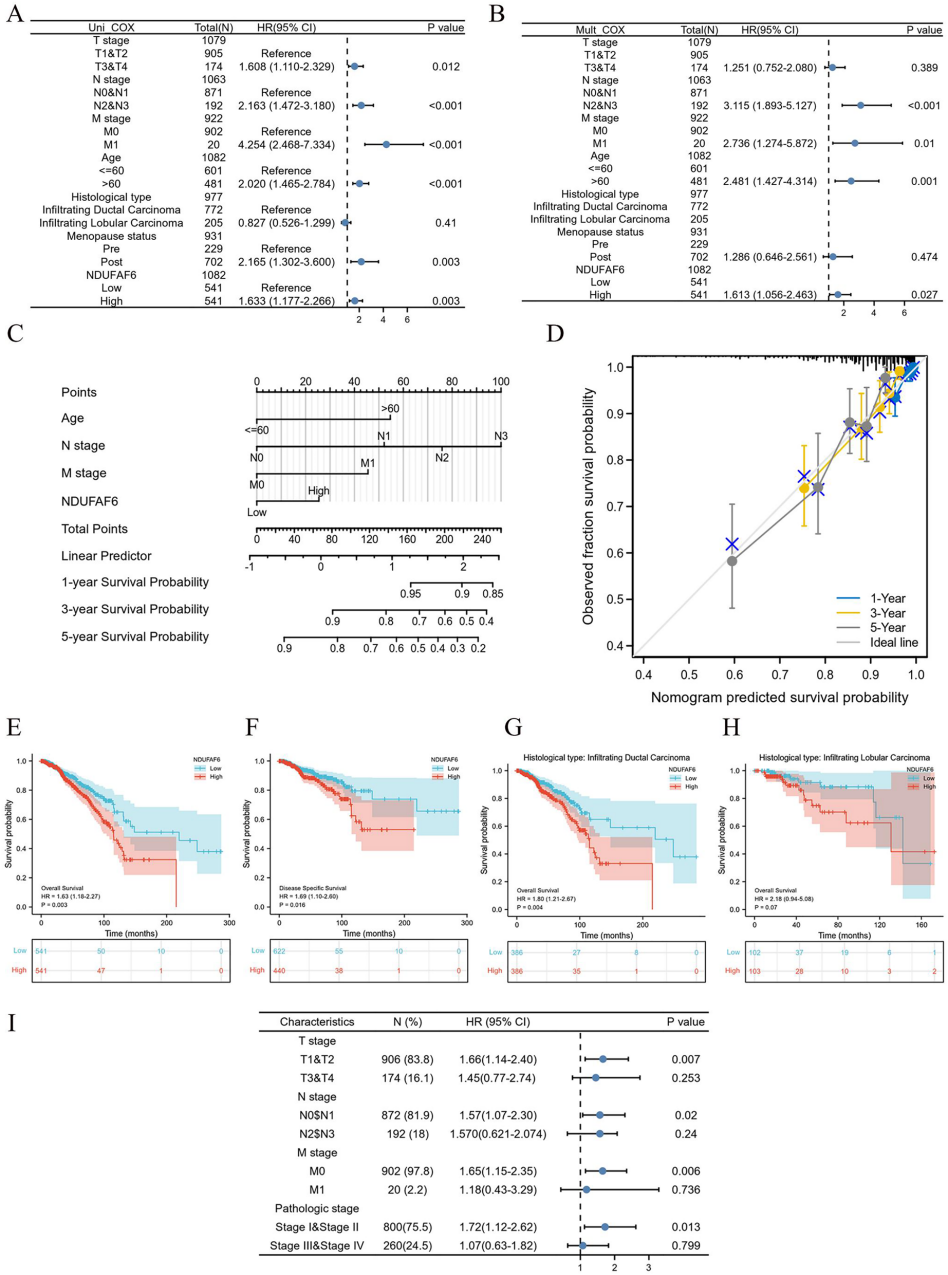
Systematic analysis of NDUFAF6 expression in breast cancer and its association with patient prognosis. **A**, **B** Display the impact of NDUFAF6 and other clinical variables on overall survival (OS) through univariate and multivariate Cox regression models. **C** Nomogram plotted based on NDUFAF6 expression, age, N stage, and M stage for patient prognosis. **D** Calibration plot validating the prognostic prediction ability of the nomogram for overall survival (OS) in breast cancer patients. **E**, **F** Kaplan–Meier survival curves reveal the relationship between NDUFAF6 expression and overall survival (OS) and disease-specific survival (DSS). **G**, **H** Relationship between NDUFAF6 expression and overall survival (OS) in different breast cancer subtypes (ductal and lobular). **I** Forest plot showcasing the impact of NDUFAF6 expression on overall survival (OS) in breast cancer patients across different TNM and pathological stages

In tumor research, identifying molecular markers associated with patient prognosis is key to understanding tumor biology and guiding clinical treatment. In this context, we further assessed the predictive value of the NDUFAF6 gene in BC. Using univariate and multivariate Cox regression models, we explored the relationship between NDUFAF6 expression and patients' overall survival (OS). The results indicated that NDUFAF6 expression and age, M stage, and N stage were identified as independent factors affecting BC OS (Fig. 4A, B).

Further nomogram evaluation revealed that as the scores for NDUFAF6, age, N stage, and M stage increased, the prognosis significantly worsened, supported by calibration curves for 1-year, 3-year, and 5-year OS predictions (Fig. 4C, D).

For a more comprehensive survival analysis, we also studied the relationship between NDUFAF6 expression and two key survival indicators in BC patients—overall survival (OS) and disease-specific survival (DSS). Kaplan–Meier survival curves showed that patients with high NDUFAF6 expression had shorter OS and DSS than those with low NDUFAF6 expression (Fig. 4E, F). In various BC subgroups, NDUFAF6 expression showed a negative trend with OS (Fig. 4G, H). Additionally, to delve deeper into the impact of NDUFAF6 on the prognosis of patients at varying stages of BC, our investigation revealed that NDUFAF6 remains a viable prognostic indicator in BC stages T1&T2, M0, as well as Stage I&Stage II. These findings were visually represented through a forest plot (Fig. 4I). Our analyses conclusively demonstrate a significant correlation between NDUFAF6 expression in breast cancer and patient prognosis.

### Differential expression of NDUFAF6 in breast cancer and its association with the NRF2 pathway

For disease-specific genes like NDUFAF6, differential expression, functional annotation, and pathway enrichment analysis provide key insights into their role in BC.

**Fig. 5:**
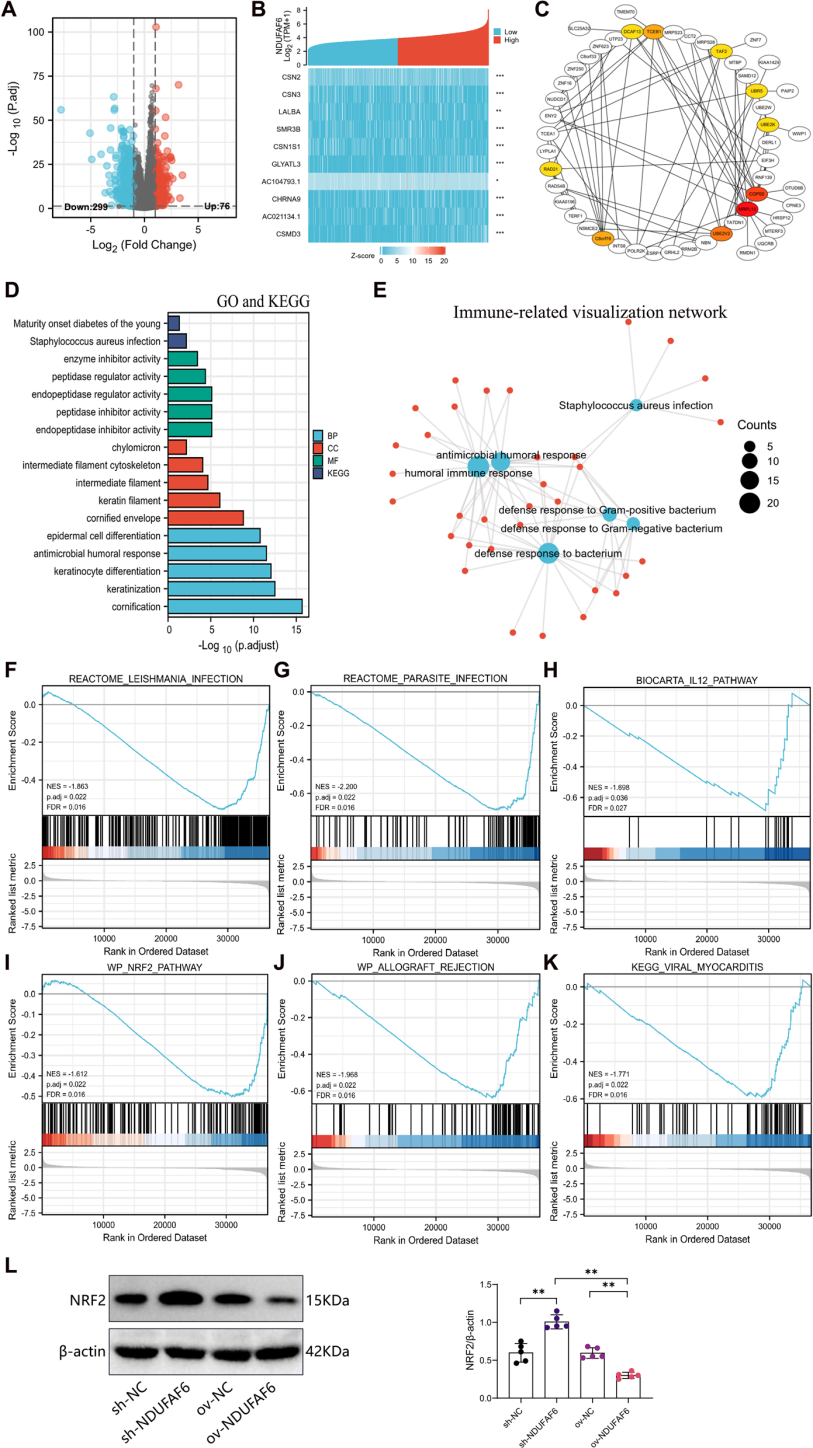
Differential expression of NDUFAF6 in breast cancer and enrichment of associated biological functions and pathways. **A** Volcano plot of DEGs between high and low NDUFAF6 expression groups, with red indicating upregulation and blue indicating downregulation. **B** Heatmap of the top 10 differentially expressed genes between high and low NDUFAF6 expression groups. **C** PPI network of NDUFAF6 and its co-expressed genes. **D**, **E** GO and KEGG enrichment analysis based on NDUFAF6 differentially expressed genes and visualization of immunity-related pathways. **F**–**K** Enrichment plots from GSEA include **F** Leishmania infection, **G** Parasite infection, **H** IL12 pathway, **I** NRF2 pathway, **J** allograft rejection, and **K** Viral myocarditis. **L** Western Blot validation of changes in NRF2 in MCF-7 cell xenografts after silencing or overexpressing NDUFAF6 (n = 5)

After an in-depth analysis of the TCGA dataset, we identified 309 differentially expressed genes (DEGs) associated with high and low expression of NDUFAF6 using the DSEeq2 tool, of which 76 were upregulated, and 229 were downregulated (Fig. 5A, B). To further decipher the functional network of NDUFAF6, we constructed its protein–protein interaction (PPI) network, highlighting 10 core genes within it (Fig. 5C). Through GO and KEGG enrichment analyses, we found that NDUFAF6 is mainly associated with biological processes and pathways such as Staphylococcus aureus infection, antimicrobial humoral response, defense responses against gram-positive and gram-negative bacteria, and humoral immune response (Fig. 5D, E). Further GSEA analysis revealed significant pathways between high and low NDUFAF6 expression groups, including Leishmania infection, parasitic infections, IL12 pathway, NRF2 pathway, allograft rejection, and viral myocarditis (Fig. 5F, K). In BC cell lines and patient samples, Nrf2 was downregulated compared to healthy breast epithelial cells [26]. Especially in TNBC patients, Nrf2 was significantly reduced compared to non-TNBC patients, potentially highlighting the importance of Nrf2's chemopreventive function [26]. To verify the association between NDUFAF6 and NRF2, we conducted experimental validations. The Western blot results revealed that increased expression of NDUFAF6 inhibits the accumulation of NRF2, whereas inhibiting NDUFAF6 expression enhances NRF2 expression (Fig. 5L).

In summary, the differential expression of NDUFAF6 in breast cancer is associated with various biological processes and pathways, especially its connection with the NRF2 pathway.

### Association of NDUFAF6 expression in breast cancer with the tumor microenvironment and immune checkpoints

The tumor microenvironment (TME) plays a crucial role in the occurrence, development, and response to treatment of tumors. Immune cell infiltration and immune checkpoints hold a special position within the TME. In order to investigate the impact of NDUFAF6 expression on the tumor microenvironment in breast cancer, we utilized the ssGSEA algorithm provided in the R package "GSVA" [27]. We conducted an analysis of immune infiltration using marker genes for 24 immune cell types reported in the literature [28]. The specific immune cell types and their corresponding marker genes can be found in Table 3.

**Table 3:** Table with the immune cells marker genes for ssGSEA

Immune cells	Marker genes
B cells	MS4A1; TCL1A; HLA-DOB; PNOC; KIAA0125; CD19; CR2; IGHG1; FCRL2; BLK; COCH; OSBPL10; IGHA1; TNFRSF17; ABCB4; BLNK; GLDC; MEF2C; IGHM; FAM30A; SPIB; BCL11A; GNG7; IGKC; CD72; MICAL3; BACH2; IGL@; CCR9; QRSL1; DTNB; HLA-DQA1; SCN3A; QRSL1; SLC15A2
T cells	PRKCQ; CD3D; CD3G; CD28; LCK; TRAT1; BCL11B; CD2; TRBC1; TRA@; ITM2A; SH2D1A; CD6; CD96; NCALD; CIMAP5; CD3E; SKAP1
T helper cells	ICOS; LRBA; ITM2A; FAM111A; PHF10; NUP107; SEC24C; NAP1L4; BATF; ASF1A; FRYL; FUSIP1; TRA@; PRA1; UBE2L3; ANP32B; DDX50; C13orf34; PPP2R5C; SLC25A12; ATF2; CD28; GOLGA8A
Tcm	CDC14A; ATM; USP9Y; PCNX; FOXP1; KLF12; ST3GAL1; INPP4B; CASP8; MLL; PCM1; RP11-74E24.2; PHC3; NFATC3; LOC202134; TIMM8A; ATF71P; REPS1; PSPC1; RPP38; HNRPH1; STX16; CYLD; SNRPN; TRAF3IP3; NEFL; POLR2J2; AQP3; CG030; PDXDC2; CLUAP1; DOCK9; CYorf15B; CREBZF; CEP68; TXK; SLC7A6; FYB; MAP3K1
Tem	TRA@; PRKY; VIL2; GDPD5; CCR2; MEFV; C7orf54; FLI1; TBC1D5; DDX17; AKT3; EWSR1; TBCD; CCR2; NFATC4; LTK
Th1 cells	IFNG; LTA; APBB2; APOD; ZBTB32; CD38; CSF2; CTLA4; CD70; DPP4; EGFL6; BST2; DUSP5; LRP8; IL22; DGKI; CCL4; DPP4; GGT1; LRRN3; SYNGR3; ATP9A; BTG3; CMAH; HBEGF; SGCB
Th2 cells	PMCH; AHI1; PTGIS; CXCR6; EVI5; IL26; MB; NEIL3; GSTA4; PHEX; SMAD2; CENPF; ANK1; ADCY1; AI582773; LAIR2; SNRPD1; MICAL2; DHFR; WDHD1; BIRC5; SLC39A4; HELSS; LIMA1; CDC25C; CD27; GATA3
TFH	CHI3L2; CXCL13; MYO7A; CHGB; ICA1; HEY1; CDK5R1; ST8SIA1; PDCD1; BLR1; KIAA1324; PVALB; TSHR; C18orf1; TOX; SLC7A10; SMAD1; POMT1; PASK; MKL2; PTPN13; KCNK5; ZNF764; MAF; MYO6; SIRPG; THADA; MAGEH1; B3GAT1; SH3TC1; HIST1H4K; STK39
Th17 cells	IL17A; IL17RA; RORC
TReg	FOXP3
CD8 T cells	CD8B; CD8A; PF4; PRR5; SF1; LIME1; DNAJB1; ARHGAP8; GZMM; SLC16A7; SFRS7; APBA2; C4orf15; LEPROTL1; ZFP36L2; GADD45A; MYST3; ZEB1; ZNF609; C12orf47; THUMPD1; VAMP2; ZNF91; ZNF22; TMC6; DNAJB1; FLT3LG; CDKN2AIP; TSC22D3; TBCC; RBM3; ABT1; C19orf6; CAMLG; PPP1R2; AES; KLF9; PRF1
Tgd	TRD@; TARP; C1orf61; TRGV9; CD160; TARP; FEZ1
Cytotoxic cells	KLRD1; KLRF1; GNLY; CTSW; KLRB1; KLRK1; NKG7; GZMH; SIGIRR; ZBTB16; RUNX3; APOL3; APBA2; WHDC1L1; DUSP2; GZMA
NK cells	LOC643313; GAGE2; ZNF747; XCL1; XCL2; AF107846; SLC30A5; NM_014114; MCM3AP; TBXA2R; CDC5L; LOC730096; FUT5; FGF18; MRC2; RP5-886K2.1; SPN; PSMD4; PRX; FZR1; ZNF205; AL080130; ZNF528; MAPRE3; BCL2; NM_017616; ARL6IP2; PDLIM4; NM_014274; LDB3; ADARB1; SMEK1; TCTN2; TINAGL1; IGFBP5; ALDH1B1; NCR1
NK CD56dim cells	KIR3DL2; SPON2; KIR2DL3; GZMB; KIR3DS1; KIR3DL1; FLJ20699; TMEPAI; IL21R; KIR2DS5; KIR2DS2; GTF3C1; KIR2DS1; EDG8
NK CD56bright cells	DUSP4; RRAD; XCL1; PLA2G6; NIBP; FOXJ1; MADD; BG255923; MPPED1; MUC3B
DC	CD209; CCL17; HSD11B1; CCL13; CCL22; PPFIBP2; NPR1
iDC	CD1B; VASH1; F13A1; MMP12; FABP4; CLEC10A; SYT17; MS4A6A; CTNS; GUCA1A; CARD9; CD1E; ABCG2; CD1A; PPARG; PAP1GAP; SLC7A8; GSTT1; NM_021941; FZD2; CSF1R; HS3ST2; CH25H; LMAN2L; SLC26A6; BLVRB; NUDT9; PREP; TM7SF4; TACSTD2; CD1C
aDC	CCL1; EBI3; INDO; LAMP3; OAS3
pDC	IL3RA
Eosinophils	IL5RA; KCNH2; TKTL1; EMR1; KCNH2; CCR3; ACACB; THBS1; GALC; RNU2; CLC; HIST1H1C; CYSLTR2; HRH4; RNASE2; CAT; LRP5L; SYNJ1; THBS4; GPR44; KBTBD11; HES1; ABHD2; TIPARP; SMPD3; MYO15B; TGIF1; RRP12; IGSF2; HES1; RCOR3; EPN2; C9orf56; SIAH1
Macrophages	MARCO; CXCL5; SCG5; SULT1C2; MSR1; CTSK; PTGDS; COLEC12; GPC4; PCOLCE2; CHIT1; KAL1; CLEC5A; ME1; DNASE2B; CCL7; FN1; CD163; GM2A; SCARB2; BCAT1; RAI14; COL8A2; APOE; CHI3L1; ATG7; CD84; FDX1; MS4A4A; SGMS1; EMP1; CYBB; CD68
Mast cells	PRG2; CTSG; TPSAB1; SLC18A2; TPSAB1; MS4A2; CPA3; TPSB2; NM_003293; GATA2; HDC; LOH11CR2A; SIGLEC6; ELA2; CMA1; PGDS; MLPH; ADCYAP1; SLC24A3; CALB2; KIT; ABCC4; PPM1H; MAOB; HPGD; SCG2; PTGS1; CEACAM8; MPO; NR0B1; LOC339524
Neutrophils	CSF3R; CYP4F3; VNN3; FPRL1; KCNJ15; MME; IL8RA; IL8RB; FCGR3B; DYSF; FCAR; CEACAM3; FPRL1; HIST1H2BC; HPSE; FLJ11151; CREB5; S100A12; TNFRSF10C; SLC22A4; KIAA0329; SLC25A37; BST1; FCAR; CEACAM3; CRISPLD2; G0S2; SIGLEC5; CD93; MGMA; ALPL; FPR1; PDE4B; LILRB2

**Fig. 6:**
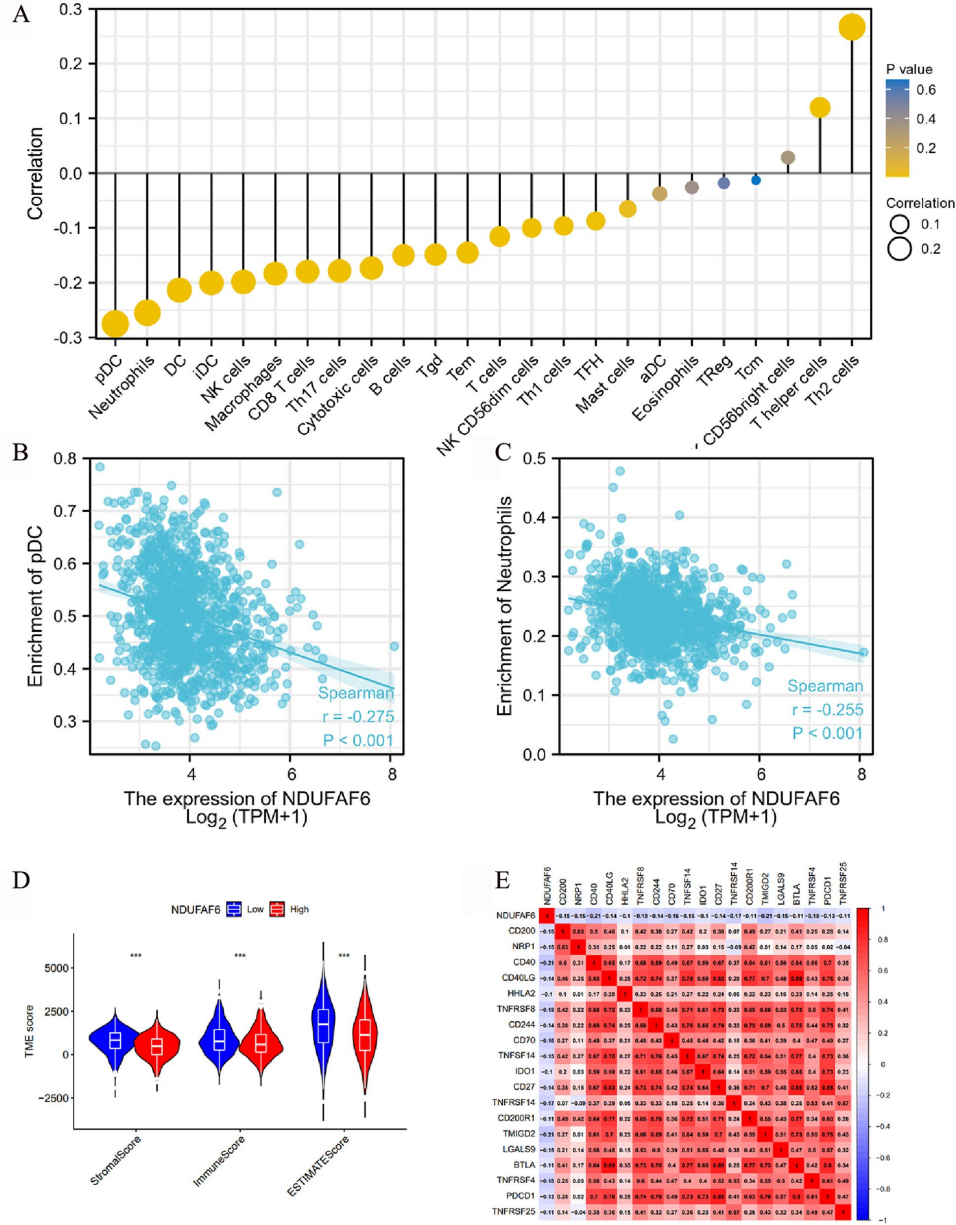
Analysis of immune infiltration cells and correlation with immune checkpoints. **A** Analysis of the correlation between the number of 24 immune cells and NDUFAF6 expression. **B** pDC cells (r = − 0.275, p < 0.001). **C** Neutrophils (r = − 0.255, p < 0.001). **D** Stromal, immune, and ESTIMATE scores. **E** Correlation between NDUFAF6 and key immune checkpoint genes

Through Spearman correlation testing, we explored the relationship between immune cell enrichment in breast cancer tissues and the expression patterns of NDUFAF6. The results showed that the expression pattern of NDUFAF6 negatively correlated with several anti-tumor immune cells, such as pDC cells and neutrophils, which play major roles in tumor suppression (Fig. 6A–C). Next, we evaluated the stromal, immune, and ESTIMATE scores in breast cancer using the ESTIMATE method, revealing a significant negative correlation with NDUFAF6 expression (Fig. 6D). Further, we investigated the relationship between NDUFAF6 and immune checkpoint genes in breast cancer, finding that the expression pattern of NDUFAF6 negatively correlated with immune checkpoint genes like CD200, PD-L1, NRP1, B7-H2, and PDCD1, providing clues to the potential role of NDUFAF6 in breast cancer immunotherapy (Fig. 6E).

### NDUFAF6 effect on breast cancer xenograft tumor growth via NRF2 pathway

PD-L1 is one of the downstream targets of the Nrf2 signaling pathway and is often expressed on the surface of tumor cells [29]. Triple-negative breast cancer (TNBC) has a high level of PD-L1, making it a potential therapeutic target [30]. Therefore, we investigated the effect of NDUFAF6 on NRF2 and its potential role in PD-L1 expression and breast cancer cell growth.

**Fig. 7:**
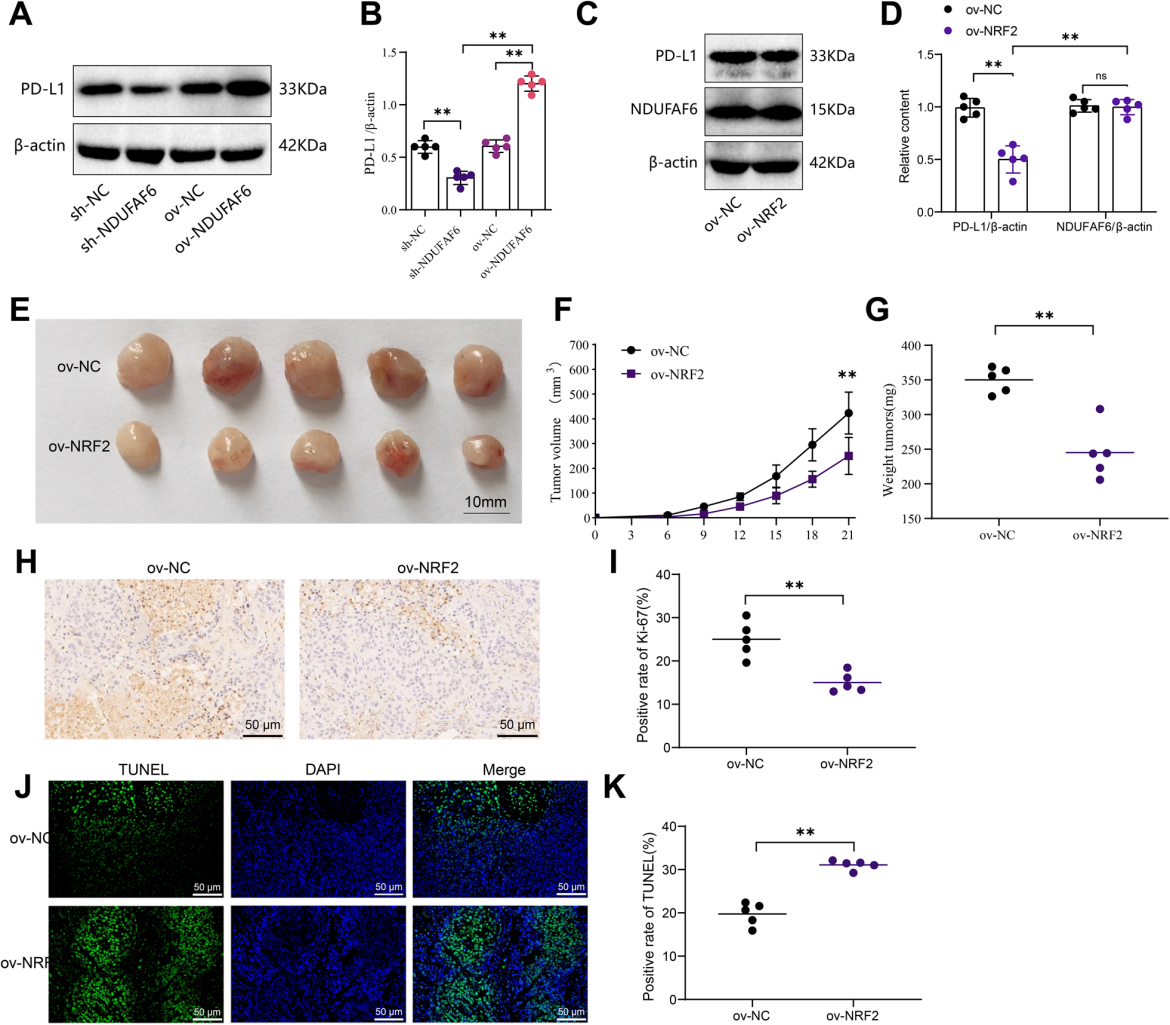
Regulatory role of NDUFAF6 on NRF2 and its impact on PD-L1 expression, cell proliferation, and apoptosis in triple-negative breast cancer. **A**, **B** Western blot was used to examine the impact of sh-NDUFAF6 and ov-NDUFAF6 treatments on the protein levels of PD-L1 in breast cancer xenografts, as well as their quantitative analysis (n = 5). **C**, **D** Western blot was performed to assess the influence of NRF2 overexpression on the protein levels of PD-L1 and NDUFAF6, along with their quantitative analysis (n = 5). **E** Physical images of xenografts formed by ov-NC group and ov-NRF2 group (n = 5). **F**, **G** Impact of NRF2 overexpression on the volume and weight of MCF-7 cell xenografts (n = 5). **H**, **I** Immunohistochemical detection of changes in the Ki-67 positivity rate in xenograft tissues and its quantitative analysis (n = 5). **J**, **K** TUNEL assay detection of the impact of NRF2 overexpression on the apoptosis rate of MCF-7 cell xenografts and its quantitative analysis (n = 5). **Indicates P < 0.01

In the mouse model of breast cancer xenografts, we observed that sh-NDUFAF6 significantly inhibited the protein level of PD-L1 in the tumor compared to sh-NC. Conversely, ov-NDUFAF6 significantly increased the protein level of PD-L1 in the tumor compared to ov-NC (Fig. 7A, B). Combining the detection of NRF2 protein expression in Fig. 5, we speculate that NDUFAF6 may promote the expression of PD-L1 by inhibiting NRF2. Further experiments demonstrated that overexpression of NRF2 significantly reduced the level of PD-L1, while there was no significant change in the protein level of NDUFAF6 (Fig. 7C, D). These results suggest that NDUFAF6 may promote PD-L1 expression by inhibiting NRF2. Additionally, overexpression of NRF2 significantly inhibited the growth of MCF-7 xenografts, as evidenced by a significant decrease in tumor volume and mass (P < 0.01) (Fig. 7E–G). Ki-67, a marker of cell proliferation, had its expression in xenograft tissues significantly regulated by NRF2, with overexpression of NRF2 leading to a significant decrease in the proportion of Ki-67 positive cells, while its overexpression resulted in a significant increase in the proportion of Ki-67 positive cells (P < 0.01) (Fig. 7H, I). Lastly, through TUNEL assays, we found that overexpression of NRF2 significantly increased the apoptosis rate of MCF-7 cell xenograft tumors (P < 0.01) (Fig. 7J, K).

In summary, the collaboration between NDUFAF6 and NRF2 promotes the expression of PD-L1 and tumor growth in breast cancer cells.

## Discussion

According to recent cancer statistics, the incidence of BC is on par with lung cancer [31, 32]. As such, BC poses a significant threat to human health. Despite recent advancements in early detection, diagnosis, and therapeutic approaches, the prognosis remains poor for patients with advanced and/or metastatic BC [33]. Numerous studies have highlighted the pivotal role of the immune system in the progression of BC [34–36]. Therefore, identifying new predictive biomarkers and options for immunotherapy is a crucial step in BC treatment.

NDUFAF6, located on chromosome 8 q22.1, encodes a mitochondrial protein containing a putative plant coenzyme domain [37]. To date, the role of NDUFAF6 in tumors remains largely uncharted. Previous research identified a novel genomic fusion event between NDUFAF6 and ARHGEF3 in prostate cancer [38]. Whether NDUFAF6 has fusion events with other genes warrants further exploration. NDUFAF6 has been suggested to be involved in the pathogenesis of hepatocellular carcinoma by controlling mitochondrial-mediated translational processes [18]. Familial pulmonary fibrosis has been linked to mutations in genes on telomeres, including NDUFAF6 [39]. Our study, utilizing multiple databases, reveals a significant upregulation of NDUFAF6 in BC. More importantly, high expression of NDUFAF6 is closely associated with a poor prognosis in BC, suggesting NDUFAF6 could emerge as a new independent predictor for BC prognosis.

Using TCGA data, we initially assessed the expression pattern of NDUFAF6 across pan-cancers, specifically in BC. The findings indicate that NDUFAF6 expression is significantly elevated in 23 types of cancers, including BC. ROC curve analysis suggests that NDUFAF6 appears to have good diagnostic accuracy (AUC = 0.931). The elevated expression levels of NDUFAF6 were consistent in the GEO databases (GSE109169 and GSE22820) and the HPA database. Additionally, clinical sample testing of BC samples was conducted; IHC analysis confirmed an elevated protein expression of NDUFAF6 compared to healthy tissues adjacent to malignant tumors.

Furthermore, we found that the expression of NDUFAF6 is closely related to ER status, menopausal status, age, PR status, N stage, PAM50, histological type, and race in BC patients. Notably, we observed that the expression level of NDUFAF6 is significantly upregulated in individuals over 60 compared to those under 60. We speculate that this gene might promote BC onset by accelerating human cellular aging. Subsequently, we assessed the relationship between NDUFAF6 levels and the prognosis of cancer patients. Evaluations of OS and DSS revealed that NDUFAF6 levels correlate with the prognosis of BC patients, making it a hazardous variable in BC. NDUFAF6 emerges as an independent predictive biomarker determined by univariate and multivariate Cox regression. The current findings suggest that NDUFAF6 plays a significant role in predicting the prognosis of BC patients, thus establishing it as a reliable biomarker.

Another key finding of our study is the association of NDUFAF6 expression with immune infiltration in BC. Moreover, a recent study proposed an association of NDUFAF6 with the pre-immune microenvironment in BC [40]. NDUFAF6 showed a negative correlation with anti-tumor immune cells like pDC cells and neutrophils, suggesting that NDUFAF6 might influence the TME, affecting BC progression and prognosis. Using the ESTIMATE algorithm, an inverse relationship was observed between NDUFAF6 and the stromal, immune, and ESTIMATE scores in BC. Interestingly, we found that the expression pattern of NDUFAF6 negatively correlates with key immune checkpoint genes, hinting at the potential role of NDUFAF6 in predicting the immunotherapeutic response in BC. In vivo experiments further revealed that NDUFAF6 could promote PD-L1 expression in breast cancer cells by inhibiting the NRF2 signaling pathway. The NRF2 signaling pathway is associated with tumor growth and metastasis in various cancers, and the expression of PD-L1 is related to immune evasion. It provides a new mechanistic explanation for the role of NDUFAF6 in BC.

Considering how to target NDUFAF6 is beneficial. Conventionally, antibodies and inhibitors are the primary strategic focus for NDUFAF6; however, each strategy has its merits. Using antibodies to target NDUFAF6 could enhance precision [41, 42], but the design and production of antibodies are costly. A small molecule inhibitor of NDUFAF6 would be a cost-effective targeting approach, but currently, there are no reports on existing NDUFAF6 inhibitors. Whether NDUFAF6 offers advantages over other BC prognostic indicators is worth exploring. Thus, future research on NDUFAF6 needs to be more in-depth and more integrated with clinical patients.

## Conclusion

**Fig. 8:**
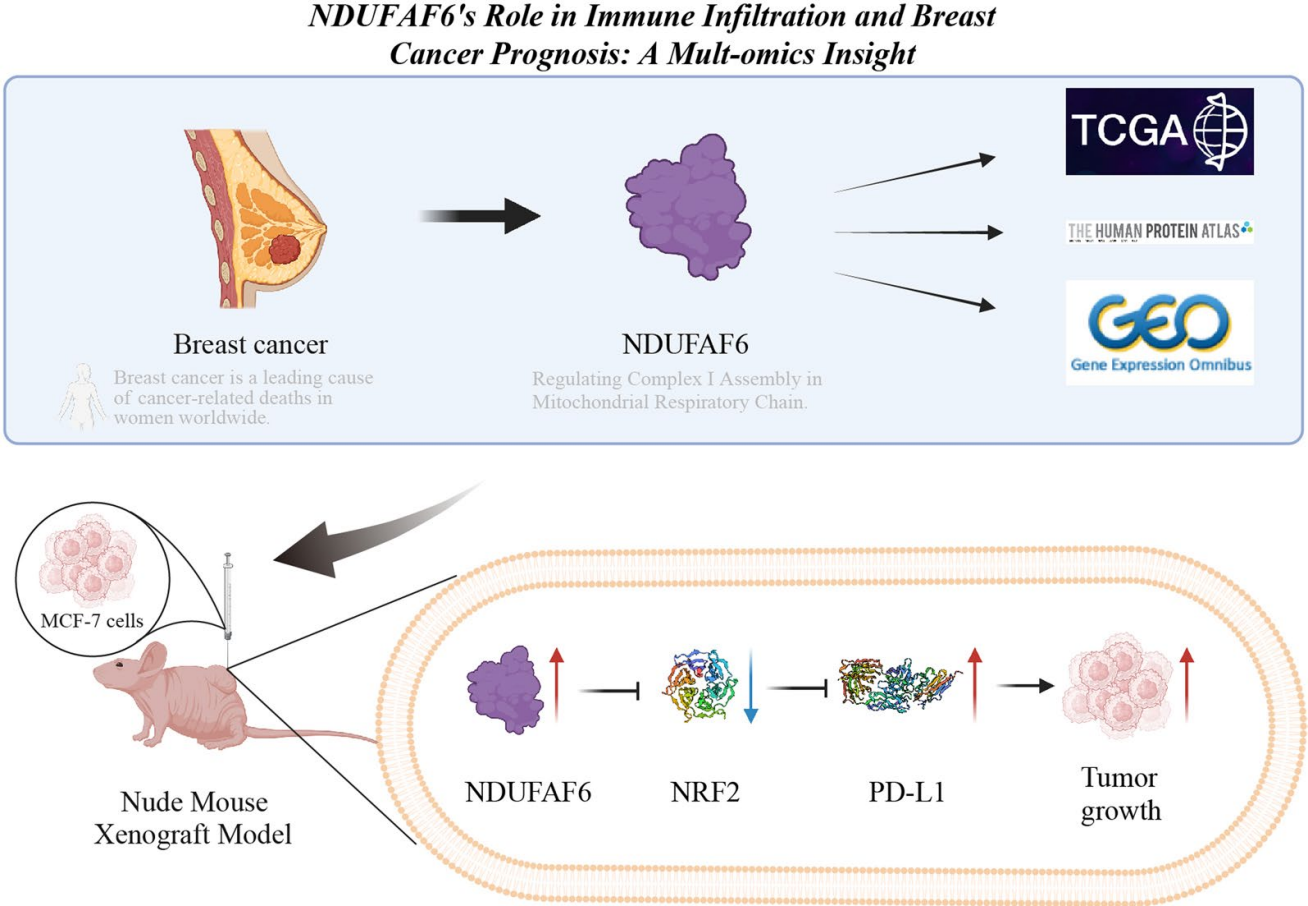
Multi-omics combined analysis reveals the mechanism by which NDUFAF6 mediates immune infiltration and subsequently affects breast cancer prognosis

In conclusion, NDUFAF6 protein expression is significantly elevated in BC patients and breast cancer cell xenograft mice. Elevated expression of NDUFAF6 is closely associated with adverse clinical pathological features and shorter survival times in BC. Through in-depth mechanistic studies, we revealed that NDUFAF6 affects immune infiltration and tumor growth in BC by inhibiting the NRF2 signaling pathway, thereby promoting PD-L1 expression in breast cancer cells (Fig. 8). This discovery provides new evidence for the pivotal role of NDUFAF6 in BC. In the future, we plan to delve deeper into the mechanistic role of NDUFAF6 in BC, especially its interactions with other signaling pathways. Additionally, we aim to explore the potential application of NDUFAF6 inhibitors in BC treatment, offering more effective therapeutic strategies for BC patients.

### Supplementary Information

**Additional file 1: Figure S1.** Differential expression of members of the NADH-ubiquinone oxidoreductase complex I in breast cancer and healthy tissues.The expression profiles of NDUFAF1 (A), NDUFAF2 (B), NDUFAF3 (C), NDUFAF4 (D), NDUFAF5 (E), NDUFAF7 (F), NDUFAF8 (G), MT-ND1 (H), MT-ND2 (I), MT-ND3 (J), MT-ND4 (K), and MT-ND5 (L) were compared between breast cancer and unmatched healthy tissue samples, as well as between breast cancer and their respective non-cancerous tissue samples.

**Additional file 2: Figure S2.** Differential expression of members of the NADH-ubiquinone oxidoreductase complex I in breast cancer and healthy tissues.The expression patterns of MT-ND6 (A), NDUFS1 (B), NDUFS2 (C), NDUFS3 (D), NDUFS4 (E), NDUFS5 (F), NDUFS6 (G), NDUFS7 (H), NDUFS8 (I), NDUFV1 (J), NDUFV2 (K), and NDUFV3 (L) were compared between breast cancer tissue samples and unmatched healthy tissue samples, as well as between breast cancer and their respective non-cancerous tissue samples.

**Additional file 3: Figure S3.** Western Blot detection of the efficiency of silencing or overexpressing NDUFAF6 in breast cancer MCF-7 cell xenografts. **Indicates P < 0.01.

## Data Availability

The datasets generated and/or analyzed during the current study are available from the corresponding author on reasonable request.
