# Supplementary figures and images for: Impact of NDUFAF6 on breast cancer prognosis: linking mitochondrial regulation to immune response and PD-L1 expression
Source: Cancer Cell Int. 2024 Mar 8;24:99. doi: 10.1186/s12935-024-03244-1 (PMC10921816; doi:10.1186/s12935-024-03244-1)

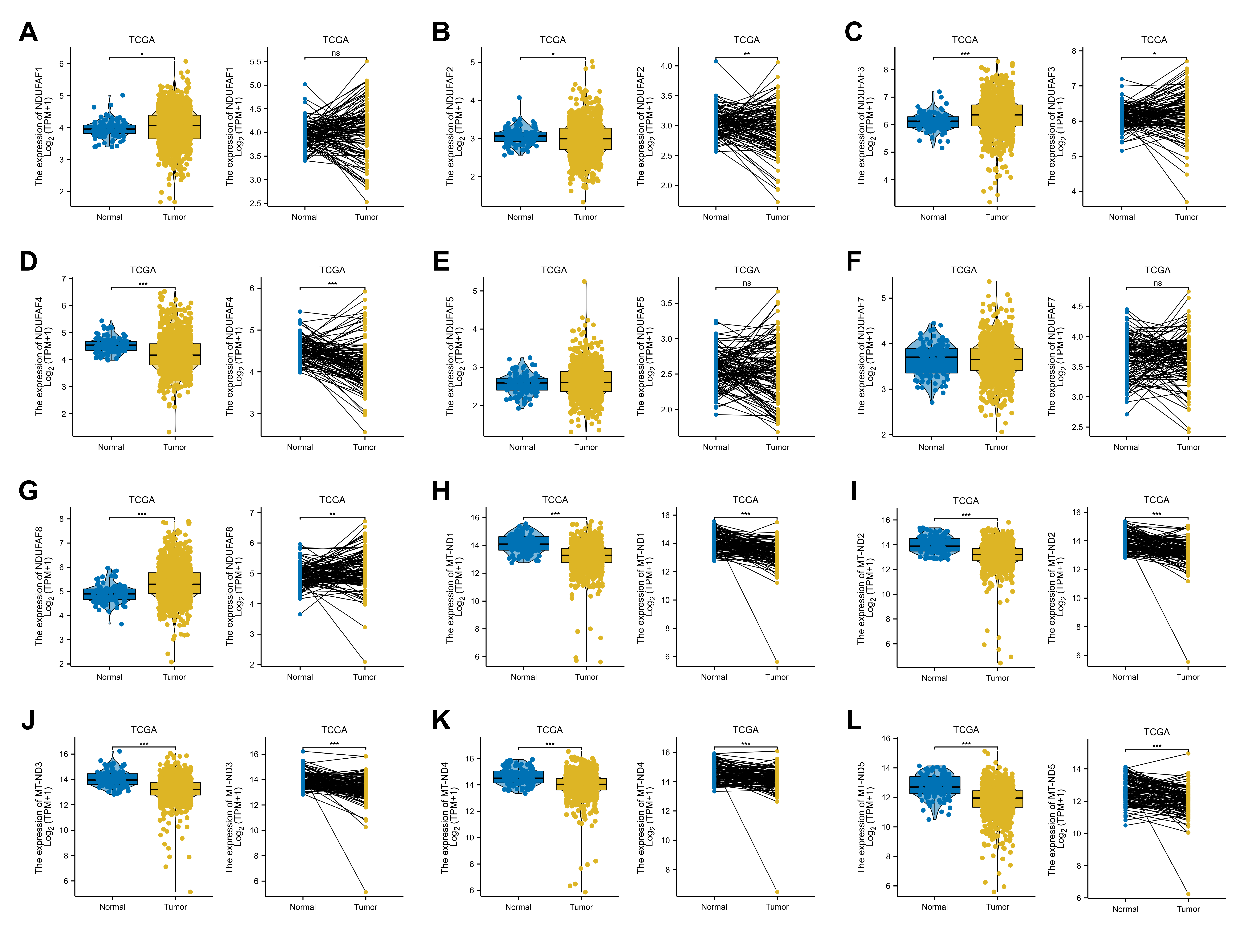

Supplement: Supplementary file 1 — Additional file 1: Figure S1. Differential expression of members of the NADH-ubiquinone oxidoreductase complex I in breast cancer and healthy tissues.The expression profiles of NDUFAF1 (A), NDUFAF2 (B), NDUFAF3 (C), NDUFAF4 (D), NDUFAF5 (E), NDUFAF7 (F), NDUFAF8 (G), MT-ND1 (H), MT-ND2 (I), MT-ND3 (J), MT-ND4 (K), and MT-ND5 (L) were compared between breast cancer and unmatched healthy tissue samples, as well as between breast cancer and their respective non-cancerous tissue samples. [file 12935_2024_3244_MOESM1_ESM.jpg]

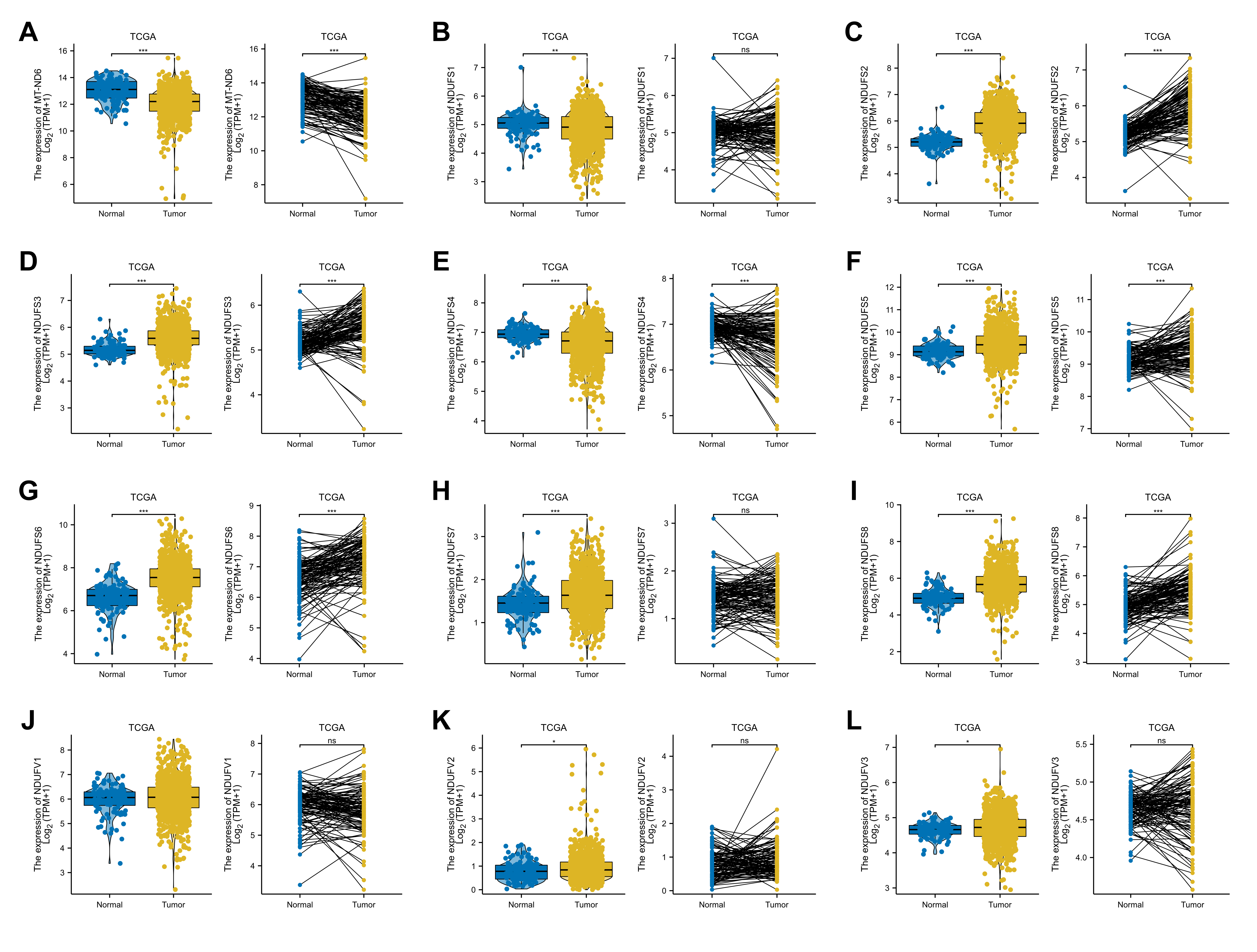

Supplement: Supplementary file 2 — Additional file 2: Figure S2. Differential expression of members of the NADH-ubiquinone oxidoreductase complex I in breast cancer and healthy tissues.The expression patterns of MT-ND6 (A), NDUFS1 (B), NDUFS2 (C), NDUFS3 (D), NDUFS4 (E), NDUFS5 (F), NDUFS6 (G), NDUFS7 (H), NDUFS8 (I), NDUFV1 (J), NDUFV2 (K), and NDUFV3 (L) were compared between breast cancer tissue samples and unmatched healthy tissue samples, as well as between breast cancer and their respective non-cancerous tissue samples. [file 12935_2024_3244_MOESM2_ESM.jpg]

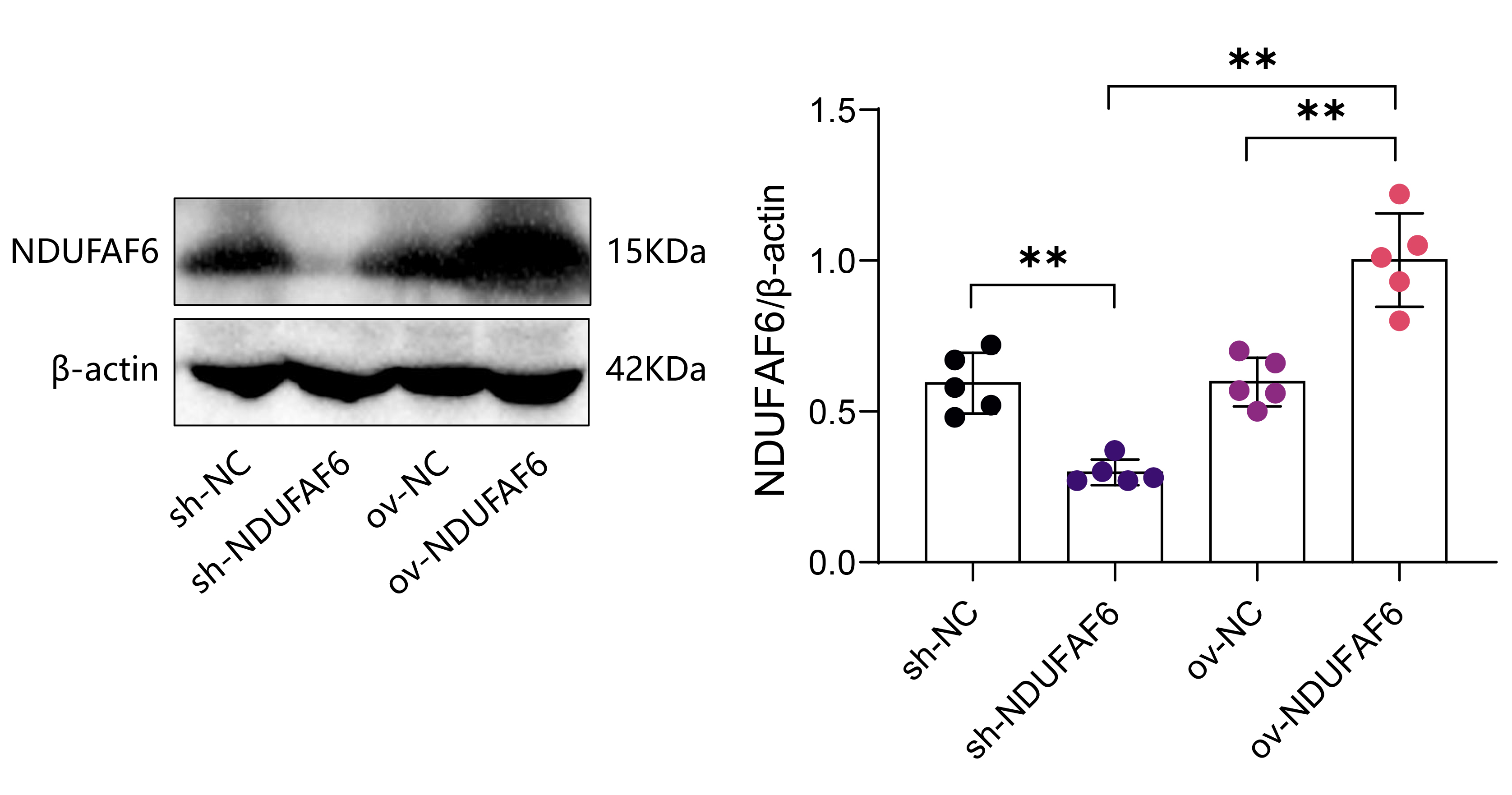

Supplement: Supplementary file 3 — Additional file 3: Figure S3. Western Blot detection of the efficiency of silencing or overexpressing NDUFAF6 in breast cancer MCF-7 cell xenografts. **Indicates P < 0.01. [file 12935_2024_3244_MOESM3_ESM.jpg]
